# 

**Published:** 1889-03

**Authors:** 


### Programme—St. Louis Dental Society.

The following papers will be read before the St. Louis Dental Society during the year 1889, and at the meetings specified:

February 19th. Dr. Geo. W. Dennis, Subject: Antiseptics and Disinfectants.
Discussion opened by Dr. Jesse E. Grosheider.

March 5th. Dr. J. E. Grosheider.......Subject: Chemistry.
Discussion opened by Dr. G. W. Dennis.

March 19th. Dr. Wm. Conrad, Subject: Dental Journals—Why Dentists Should Take and Read Them.
Discussion opened by Dr. J. W. Wick.

April 2d. Dr. H. H. Keith .....Subject: Painless Dentistry.
Discussion opened by Dr. M. C. McNamara.

April 16th. Dr. J. G. Harper.....Subject: Dental Medicine.
Discussion opened by Dr. J. W. Whipple.

May 7th. Dr. A. J. Prosser ......Subject: General Practice.
Discussion opened by Dr. Geo. Robitoy.

May 21st. Dr. J. B. Newby.........Subject: Thorough versus Quick Operations.
Discussion opened by Dr. Geo. A. Bowman.

June 4th. Dr. J. W. Whipple......Subject: Cements—Their Virtues and Faults.
Discussion opened by Dr H. H. Keith.

June 18th. Dr. A. H. Fuller ..........Subject: Conditions Affecting Practice.
Discussion opened by Dr. Henry Fisher.

July 2d. Dr. Geo. Robitoy........Subject: Cases in Practice.
Discussion opened by Dr. J. B. Vernon.

September 17th. Dr. Henry Fisher......Subject: Filling Materials and their Place in Practice.
Discussion opened by Dr. W. N. Morrison.

October 1st. Dr. J. H. Spalding..........Subject: Calcareous Deposits on the Teeth.
Discussion opened by Dr. ——

October 15th. Dr. Wm. N. Morrison .....Subject: Planting Teeth.
Discussion opened by Dr. A. H. Fuller.

November 5th. Dr. J. J. R. Patrick......Subject: The Dental Pulp and the Peridental Membrane.
Discussion opened by Dr. W. H. Eames

November 19th. Dr. W. H. Eames............Subject: Dental Literature.
Discussion opened by Dr. C. W. Spalding.

December 3d. Dr. C. W. Spalding...Subject to be announced.
Discussion opened by Dr. J. J. R. Patrick.

December 17th. Dr. Geo. A. Bowman........Subject: Dental Ethics.
Discussion opened by Dr. J. B. Newby.

### THE DENTAL REGISTER,

A MONTHLY MAGAZINE DEVOTED TO THE INTERESTS OF THE

### DENTAL PROFESSION.

*Terms Reduced to* $2.00 *Per Year. Single Copies,* 25 *Cents.*

### EDITED BY PROF. J. TAFT, D.D.S.

—AND—

### Published by SAM'L A. CROCKER & CO., Ohio Dental and Surgical Depot.

The volume commences in January and closes in December.

The REGISTER being issued monthly is always fresh, therefore Indispensable to the practitioner who desires to keep posted up with the new inventions and improvements which are daily developed. Neither expense nor effort will be spared to give value and variety to its contents. Articles will be illustrated with well executed engravings whenever they will add to the interest or assist to explanation.

Its extensive circulation and frequency of issue make it one of the BEST DENTAL ADVERTISING MEDIUMS in the United States.

All subscriptions must begin with January or July.

Local or special notices, five lines or less, will be inserted for $3 each insertion; over five lines, 50 cts. per line.

All advertising bills payable in advance, or at furthest, after the issue of the first number containing the advertisement. All transient advertisements to be paid for in advance.

☞CONTRIBUTIONS SOLICITED.

Exclusively Editorial Communications should be addressed to the Editor.

The latest number of the REGISTER may be ordered with or without other goods at any time, and all letters should be addressed to

**SAM'L A. CROCKER & CO., Ohio Dental and Surgical Depot, Cincinnati, O.**

